# Variation, Variability, and the Origin of the Avian Endocranium: Insights from the Anatomy of *Alioramus altai* (Theropoda: Tyrannosauroidea)

**DOI:** 10.1371/journal.pone.0023393

**Published:** 2011-08-10

**Authors:** Gabe S. Bever, Stephen L. Brusatte, Amy M. Balanoff, Mark A. Norell

**Affiliations:** 1 Department of Geology and Geophysics, Yale University, New Haven, Connecticut, United States of America; 2 Division of Paleontology, American Museum of Natural History, New York, New York, United States of America; 3 Department of Earth and Environmental Sciences, Columbia University, New York, New York, United States of America; University of Maryland, United States of America

## Abstract

The internal braincase anatomy of the holotype of *Alioramus altai*, a relatively small-bodied tyrannosauroid from the Late Cretaceous of Mongolia, was studied using high-resolution computed tomography. A number of derived characters strengthen the diagnosis of this taxon as both a tyrannosauroid and a unique, new species (e.g., endocranial position of the gasserian ganglion, internal ramification of the facial nerve). Also present are features intermediate between the basal theropod and avialan conditions that optimize as the ancestral condition for Coelurosauria—a diverse group of derived theropods that includes modern birds. The expression of several primitive theropod features as derived character states within Tyrannosauroidea establishes previously unrecognized evolutionary complexity and morphological plasticity at the base of Coelurosauria. It also demonstrates the critical role heterochrony may have played in driving patterns of endocranial variability within the group and potentially reveals stages in the evolution of neuroanatomical development that could not be inferred based solely on developmental observations of the major archosaurian crown clades. We discuss the integration of paleontology with variability studies, especially as applied to the nature of morphological transformations along the phylogenetically long branches that tend to separate the crown clades of major vertebrate groups.

## Introduction

The potential of developmental systems to produce morphological variation (i.e., variability) is not static but changes through ontogeny and phylogeny [1], [2]. This dynamism has important consequences not only for evolutionary mechanics and evolvability [1], [3] but also for the practical ability of biologists to precisely infer historical patterns of morphological change. The vertebrate neurocranium, for example, develops early in skeletal ontogeny, probably in response to the structural needs of the rapidly developing central nervous system [4]. This accelerated early growth is followed by a long period of negative allometry where ossification of the cartilaginous neurocranium and otic capsules and the subsequent maturation of associated ossified structures are largely delayed relative to that of other cranial partitions. The probability that in any given specimen the expression of braincase features is influenced by one of the many possible types of variation thus differs from that of other skeletal modules, even if the specimen is in a late stage of postnatal development [5]. Considering the importance of this region (both as the skeletal seat of the brain and primary sense organs and as a source of phylogenetically informative characters), the critical role paleontology plays in breaking up the long periods of evolutionary time often separating major crown clades, and the fact that any study of the fossil record likely requires comparing specimens of different ontogenetic ages, the potential implications of dynamic variability are obvious and significant. Grasping these implications requires a better understanding of developmental dynamics in extant lineages. It also requires the careful study of those extinct clades occupying critical positions on the tree, whose fossil record includes specimens with a diverse taxonomic and ontogenetic distribution. Tyrannosauroids are beginning to be one such clade [6].

While not implying directionality to the tree, the position of Tyrannosauroidea as a basal coelurosaur lineage [7]–[12] makes them a group where one would at least hope to find features intermediate between plesiomorphic states present in non-coelurosaurian theropods and highly derived morphologies characterizing maniraptoran lineages close to the crown (i.e., the origin of Aves, sensu [13]). The braincase of *Tyrannosaurus rex* long has been a subject of anatomical interest (e.g., [14]–[17]), but detailed comparative studies of endocranial anatomy among Tyrannosauroidea, especially using modern tools of anatomical visualization such as high-resolution X-ray computed tomography (CT), has only recently begun. This is true despite the fact that braincases are known for many tyrannosauroid species [18]. Several of these specimens are established as reflecting different points along the tyrannosaur growth curve [19], [20]. Recently, important contributions on this subject were published [21], [22] based largely on specimens of *Tyrannosaurus*, *Gorgosaurus libratus*, and the taxonomically controversial ‘*Nanotyrannus’ lancensis* specimen (Cleveland Museum of Natural History [CMNH] 7541, probably a juvenile *Tyrannosaurus* [23]) as well as several recently discovered taxa. These studies are critical in establish a comparative framework to which new specimens can be added and initial hypotheses of phylogenetic and ontogenetic transformations tested.

Our goal is to further address phylogenetic and ontogenetic variability in tyrannosauroids by describing the endocranial anatomy of the ontogenetically young holotype of *Alioramus altai*—a new tyrannosauroid from the Late Cretaceous of Mongolia that is nested deep within Tyrannosauridae as a close relative of the *Daspletosaurus torosus*-*Tarbosaurus bataar*-*Tyrannosaurus* clade [24]. This position places it within the *Gorgosaurus*-*Tyrannosaurus* phylogenetic bracket (equivalent to Tyrannosauridae) established by [21]. A detailed description of the well-preserved braincase will be published elsewhere. The purpose of this paper is to describe the most salient features of the internal braincase anatomy that have direct bearing on phylogenetic transformations within Tyrannosauroidea and that provide insights into the origin and early evolutionary history of coelurosaurian neuroanatomy. Discussion of these features will then be used as a springboard to address wider, more theoretical issues regarding the dynamics of morphological variation and the interpretation of evolutionary transformations in a phylogenetic context.

## Results

### General observations

**Figure 1:**
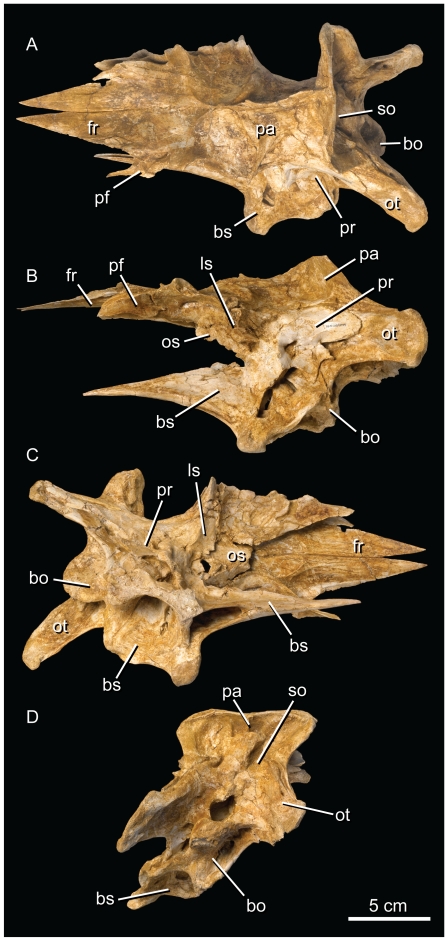
The braincase of *Alioramus altai* (IGM 100/1844) (modified from fig. 2 of [**22**]). Dorsal (A), left lateral (B), ventral (C), and occipital (D) views. bo, basioccipital; bs, basisphenoid; ls, laterosphenoid; os, orbitosphenoid; ot, otoccipital; pa, parietal; pf, prefrontal; pr, prootic; so, supraoccipital.

**Figure 2:**
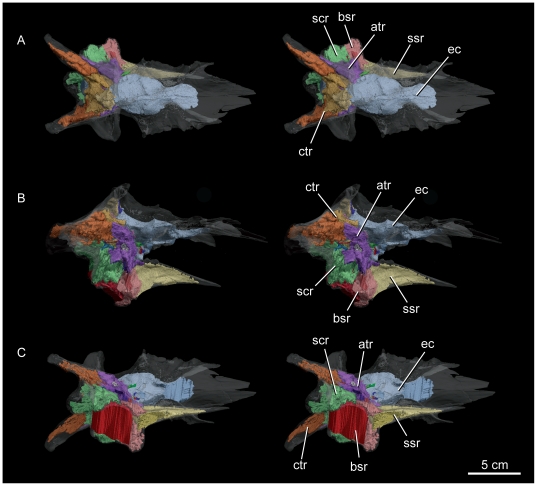
Stereopairs of the articulated braincase of *Alioramus altai* (IGM 100/1844). Dorsal (A), right lateral (B), and ventral (C) views. The images are reconstructed from the CT data with the bones rendered transparent to show the internal structures examined in this study. atr, anterior tympanic recess; bsr, basisphenoid recess; ctr, caudal tympanic recess; ec, endocranial cavity; scr, subcondylar recess; ssr, subsellar recess.

The holotype of *Alioramus altai* (Institute of Geology, Mongolian Academy of Sciences [IGM] 100/1844) was collected at Tsaagan Khushuu, Nemegt Formation (Maastrichtian) during the Mongolian Academy of Sciences-American Museum of Natural History 2001 expedition. It is estimated to represent a nine-year-old juvenile/subadult based on a count of lines of arrested growth [24]. The partial skeleton has a nearly complete braincase preserving all expected neurocranial and dermal elements except the ethmoid complex (Fig. 1). This midline element was present in life—but perhaps only as a cartilaginous structure—based on a clear frontal scar that marks the contact of the ethmoid ring with the dermal skull roof and delimits the rostrocaudal and mediolateral extent of the olfactory bulbs [25]. The braincase exhibits some clockwise distortion around the long axis of the skull that is independent of the overlying dermal roof, but overall, it is remarkable in its preservational quality, especially for a relatively small-bodied tyrannosauroid. A high-resolution CT scan resulted in remarkable contrast between matrix and bone, allowing a detailed study of the internal braincase anatomy. This anatomy includes the conformation of the endocranial cavity, the cranial nerve roots and venous sinuses, the osseous labyrinth of the inner ear, and the extensive system of internal pneumatic sinuses that invest nearly all of the neurocranial elements (Fig. 2). Descriptive observations are based on a cranial orientation in which the lateral semicircular canal is held horizontally. This configuration results in the head being held in a slightly downturned position in agreement with *Gorgosaurus* and *Tyrannosaurus* and in marked contrast to tyrannosauroid outgroups [21].

**Figure 3:**
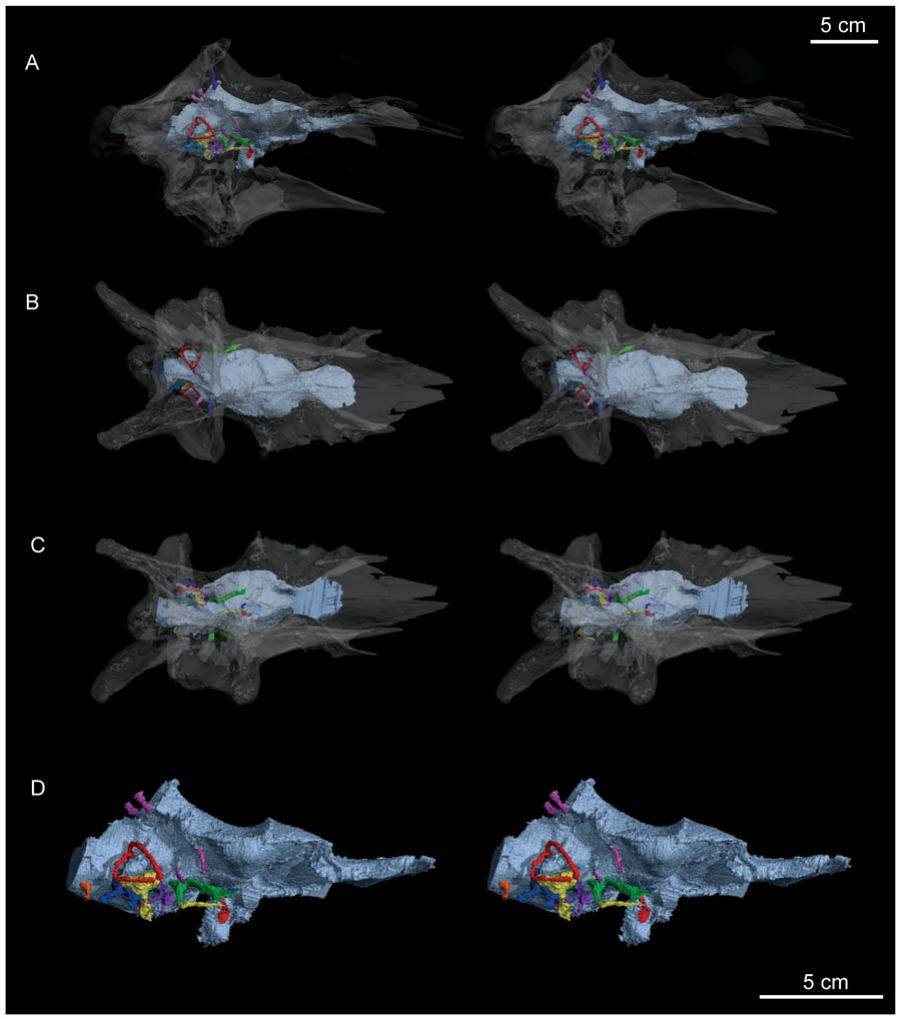
Stereopairs of the braincase of *Alioramus altai* (IGM 100/1844) rendered transparent. This rendering reveals casts of the endocranial cavity, cranial nerve roots, selected vasculature, and the inner ear in dorsal (A), right lateral (B), and ventral (C) views, and the endocast with labeled structures in right lateral view (D). The upper right scale bar refers to A–C.

**Figure 4:**
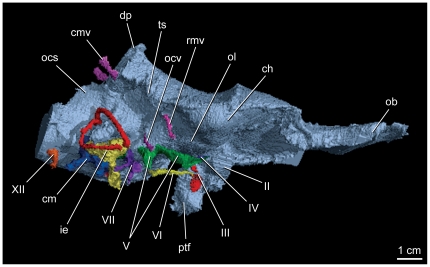
Right lateral view of labeled endocranial casts of *Alioramus altai* (IGM 100/1844). These casts include the endocranial cavity, cranial nerve roots, selected vasculature, and inner ear. II, optic canal; III, oculomotor canal; IV, trochlear canal; V, trigeminal canals; VI, abducens canal; VII, facial canals; XII, hypoglossal canal; cm, cavum metoticum, includes lateral paths of glossopharyngeal and vagus nerves; cmv, caudal middle cerebral vein canal; ch, cerebral hemisphere; dp, dorsal peak (dural sinus); ie, inner ear; ob, olfactory bulb; ocs, occipital sinus; ocv, orbitocerebral vein; ol, optic lobe; ptf, pituitary fossa; rmv, rostral middle cerebral vein canal.

As in other tyrannosauroids, the endocast is long, narrow, and apomorphically lacks a strong midbrain angle (Figs. 3, 4). Dural sinuses cover the hind- and midbrain regions and include a dorsal peak whose prominence is matched only by an adult specimen of *Tyrannosaurus rex* (American Museum of Natural History, Fossil Fishes Amphibians, Reptiles and Birds 5117 [14]). A prominent floccular lobe (cerebellar auricle) extends caudolaterally from the cerebellar region of the hindbrain to lie between the semicircular canals of the inner ear. This tabular-shaped cavity is relatively large compared to the same structure in *Tyrannosaurus*, *Gorgosaurus*, and ‘*Nanotyrannus’* and more closely resembles the floccular cavity of *Tarbosaurus* [21], [26], [27]. The path of the rostral middle cerebral vein is somewhat unclear, but it did not penetrate the laterosphenoid through its own canal as in *Gorgosaurus* and *Tyrannosaurus* [21]. It either exited with a branch of the trigeminal nerve (cranial nerve [cn] V), which is plesiomorphic for Theropoda, or through the prootic; either position would be derived within Tyrannosauroidea. Rostral to the transverse sinus is a pair of small but distinctive ventrolateral swellings interpreted to be the optic lobes. The development and position of these structures, in combination with a moderate expansion of the cerebral hemispheres, supports the hypothesis that the exaggerated cerebral expansion resulting in the caudolateral displacement of the optic lobes characteristic of avialans and other maniraptorans began at the base of Coelurosauria [17]. The olfactory tracts are extremely short and broad and do not support the tentative idea of Witmer and Ridgely [21] that broad tracts in *T. rex* and CMNH 7541 relate directly to their apomorphically broad and short skulls, because the skull of *Alioramus altai* is relatively narrow and long (it is the proportionally longest skull, compared to height, of any known tyrannosauroid specimen). The large olfactory bulbs compare closely to those of other tyrannosauroids [21], [22], [25], [28].

### Cranial nerve roots

**Figure 5:**
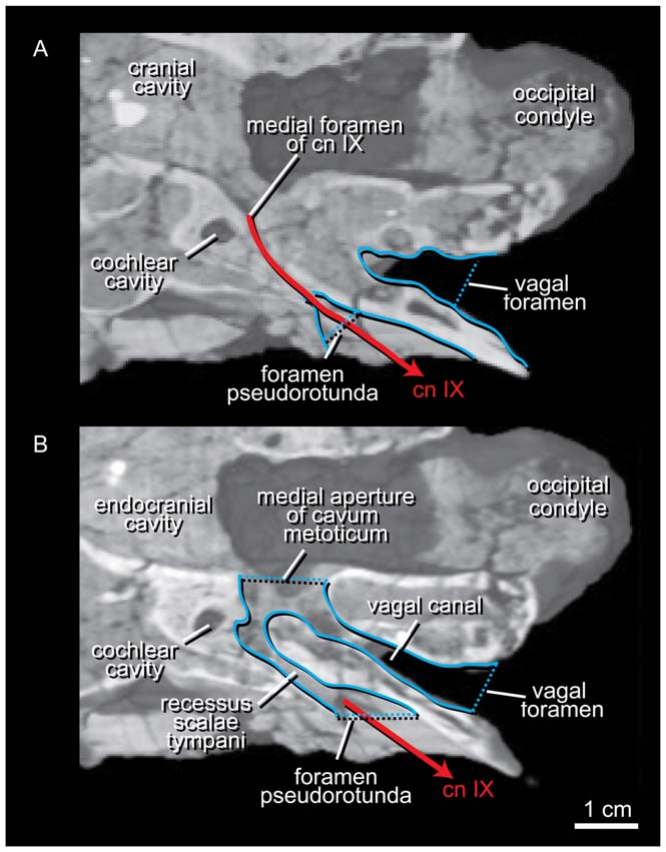
Two horizontal slices through the left cavum metoticum of *Alioramus altai* (IGM 100/1844). The slices sample the upper (A) and middle (B) regions of this space. The glossopharyngeal nerve (cn XI) enters the recessus scalae tympani through a foramen that is independent of the medial aperture of the cavum metoticum. Rostral is to the left.

The cranial nerve roots in IGM 100/1844 exhibit a basically conservative pattern with a few notable exceptions. The glossopharyngeal nerve (cn IX) exits the endocranial cavity through a separate opening in the otoccipital (fused exoccipital and opisthotic) caudal to the cochlear branch of the vestibulocochlear nerve (cn VIII) and rostral to the medial aperture of the cavum metoticum (Fig. 5). The small canal runs a short distance caudolaterally and penetrates the rostromedial wall of the recessus scalae tympani. There is no caudal path for this nerve through the pneumatized crista tuberalis to the vagal canal, so it appears the nerve maintains a rostral position and exits the braincase laterally through the foramen pseudorotunda. A rostral path for cn IX through the recessus scalae tympani is known in other tyrannosauroid and some non-tyrannosauroid theropods (21, 29), but it is unclear whether any of these taxa also express the independent medial canal of IGM 100/1844 or if their glossopharyngeal nerves communicate with the recessus scalae tympani through the medial aperture of the cavum metoticum. An independent medial canal through the rostromedial margin of the recessus scalae tympani is tentatively considered an autapomorphy of *Alioramus altai*.

**Figure 6:**
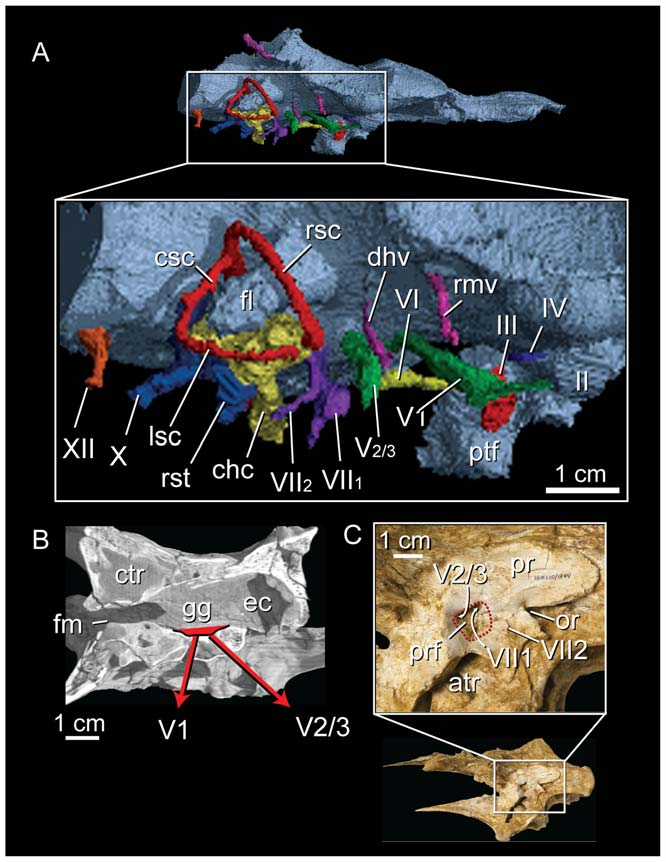
Morphology of the cranial nerve roots in *Alioramus altai* (IGM 100/1844). (A) Digital reconstruction of the cranial nerve roots in right dorsolateral view. (B) Horizontal CT slice showing the position of the gasserian ganglion and the split paths of the ophthalmic and maxillomandibular branches of the trigeminal nerve through the braincase. C) External view of the left lateral surface of the braincase showing the position of the prootic fossa, which accepts the lateral opening of the maxillomandibular branch of the trigeminal nerve and the palatine branch of the facial nerve. II, optic canal; III, oculomotor canal; IV, trochlear canal; V_1_, ophthalmic ramus of the trigeminal canal; V_2/3_, maxillomandibular ramus of the trigeminal canal; VI, abducens canal; VII_1_, palatine ramus of facial canal; VII_2_, hypoglossal ramus of facial canal; X, vagal canal; XII, hypoglossal canal; atr, anterior tympanic recess; chc, cochlear canal; csc, caudal semicircular canal; cts, caudal tympanic sinus; dhv, dorsal head vein canal; fl, floccular canal; fm, foramen magnum; gg, fossa housing gasserian ganglion; lsc, lateral semicircular canal; or, otic recess; ptf, pituitary fossa; rsc, rostral semicircular canal; rmv, rostral middle cerebral vein canal; rst, recessus scalae tympani.

The gasserian ganglion lies fully within the endocranial cavity, so that the ophthalmic (cn V_1_) and maxillomandibular (cn V_2-3_) branches of the trigeminal nerve (cn V) exit the cavity separately (Fig. 6). This is a derived feature shared with avialans, but because a single trigeminal opening characterizes the other known coelurosaur groups, paired canals can be considered an ambiguous tyrannosauroid synapomorphy [16], [21], [22], [30], [31].

A character in IGM 100/1844 currently considered an unambiguous tyrannosaur synapomorphy is the presence of a lateral fossa in the prootic that houses the external foramina of the maxillomandibular canal of the trigeminal nerve and the canal for the facial nerve (cn VII) (Fig. 6) [21], [22], [31]. The presence of this lateral fossa in the basal tyrannosauroids *Dilong paradoxus* and *Guanlong wucaii* is currently unclear [6], [32], [33]. Its expression may be diagnostic of a more exclusive clade within Tyrannosauroidea, perhaps Tyrannosauridae. The narrowing of this fossa into a foramen may be a late-stage ontogenetic transformation in tyrannosaurids [31], and the more fossa-like morphology expressed in IGM 100/1844 attests to its relative immaturity. To be precise, in IGM 100/1844 the fossa houses only the canal transmitting the palatine ramus of the facial nerve (cn VII_1_) because the geniculate ganglion was fully enclosed inside the prootic. This internal position results in separate canals for the palatine and hyomandibular branches (cn VII_2_) of the facial nerve, with the hyomandibular ramus exiting the braincase behind the prootic fossa near the otic recess. This is autapomorphic in *Alioramus altai*, as theropods (including all other tyrannosauroids) retain the highly conservative position of the geniculate ganglion within the postnatal remnant of the cranioquadrate space. When osseous signatures of the theropod palatine and hyomandibular nerves are present, they generally take the form of grooves on the lateral surface of the prootic (e.g., [31], [34]).

**Figure 7:**
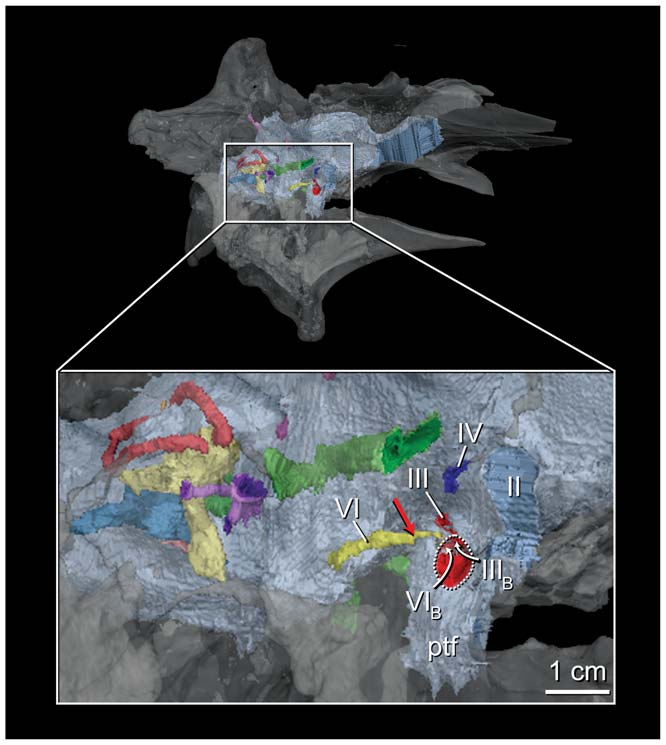
The diencephalic region of the braincase and endocranial cast of *Alioramus altai* (IGM 100/1844). The braincase is rendered in right anteroventrolateral view shows the position of the sinus cavernosus. The red arrow shows the point at which the abducens nerve enters the pituitary fossa. Dotted line delimits the sinus cavernosus as expressed on the external surface of the braincase. II, optic canal; III oculomotor canal, III_B_, position of external opening of oculomotor canal within sinus cavernosus; IV, trochlear canal; VI, abducens canal; VI_B_, position of external opening of oculomotor canal within sinus cavernosus; ptf, pituitary fossa.

Significant variation involving the cranial nerve roots is found in the area surrounding the infundibulum where IGM 100/1844 retains a plesiomorphic sinus cavernosus (Fig. 7). This sinus lies in the lateral margin of the pituitary fossa and clearly accepted the oculomotor (cn III) and abducens nerves (cn VI) and their associated vasculature. It probably also transmitted the cranial branches of the carotid arteries [35]. This is the common morphology in archosaurs generally, and in non-coelurosaurian theropods, but was previously unknown in coelurosaurs, including other tyrannosauroids [21], [29], [35]–[37]. The derived condition, retained in the crown, and expressed in all other coelurosaurs preserving this region, is the absence of a sinus cavernosus with the abducens nerve positioned lateral to the pituitary fossa [21]. The abducens canal in CMNH 7541 approximates the lateral margin of the pituitary fossa, tempting Witmer and Ridgely [21] to score the specimen as exhibiting the plesiomorphic condition. They refrained, regarding the situation as reflecting a transformation of the derived coelurosaurian morphology or an artifact of postmortem damage to the skull. The configuration in IGM 100/1844 is nearly identical to that of non-coelurosaurian theropods (e.g., *Majungasaurus crenatissimus* [29]), although the point at which the abducens nerve enters the pituitary fossa may lie closer to the lateral margin of the fossa than in non-coelurosaurian theropods. This more lateral position may reflect a secondary invasion of the pituitary fossa by the abducens nerve, but it also may reflect an intermediate state in the transition from the plesiomorphic theropod condition (abducens passes through the pituitary fossa) and the one expressed in other coelurosaurs (abducens passes lateral to the fossa), including other tyrannosauroids. Regardless of the interpretation, the abducens nerve passed through the pituitary fossa in IGM 100/1844 making the presence of a sinus cavernosus a variable morphology among tyrannosaurs.

### Osseous labyrinth

**Figure 8:**
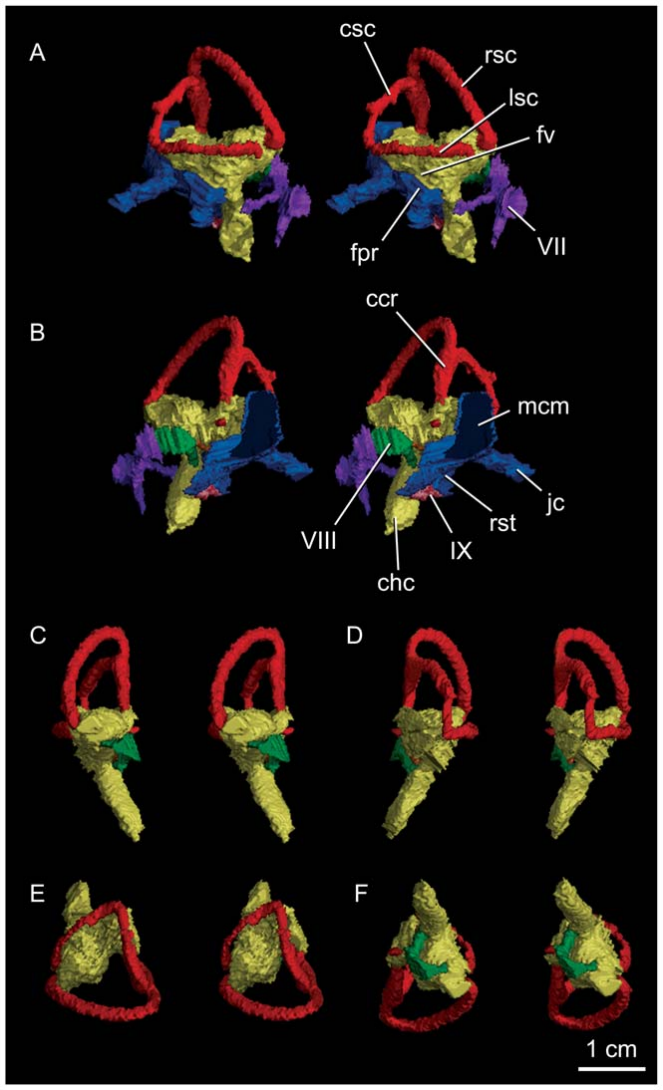
Stereopairs of the right osseous labyrinth of *Alioramus altai* (IGM 100/1844). The renderings are in lateral (A), medial (B), rostral (C), caudal (D), dorsal (E), and ventral (F) views. Reconstruction of the facial canal (purple), cavum metoticum (blue), and the independent medial path of the glossopharyngeal nerve (orange) are included in the medial and lateral views. VII, facial canals; VIII vestibulocochlear canals; IX, glossopharyngeal canal; ccr, common crus; chc, cochlear canal; csc, caudal semicircular canal; fpr, fenestra pseudorotunda; fv, fenestra vestibuli; jc, vagal canal; lsc, lateral semicircular canal; rsc, rostral semicircular canal.

The complete osseous labyrinth of IGM 100/1844 (Fig. 8) is similar to that of other tyrannosauroids in possessing an elongate rostral semicircular canal whose caudodorsal expansion and resultant twisting of the common crus is intermediate between that of non-coelurosaurian theropods and the exaggerated condition of maniraptorans [21], [38]. In contrast, the rostral expansion of this canal is restricted in IGM 100/1844 and results in the common crus retaining the plesiomorphic vertical orientation. This morphology is shared with non-coelurosaurian theropods and *Gorgosaurus*, but differs from the strongly angled common crus of *T. rex*, CMNH 7541, and maniraptorans. The lateral canal of IGM 100/1844 is not strongly bowed as in other tyrannosauroids and maniraptorans but exhibits the relatively straight orientation and strong caudal hook characteristic of non-coelurosaurian theropods. The osseous labyrinth of IGM 100/1844 is congruent with the hypothesis of [21] that a long, slender, and straight cochlear cavity is synapomorphic for tyrannosauroids.

### Neurocranial pneumaticity

The neurocranial elements of IGM 100/1844 are extensively pneumatized and house the five sinus recesses typical of tyrannosauroids (Fig. 2). The morphology of the internal cavities associated with these recesses is also conservative, again with a few notable exceptions. For example, the rostral tympanic sinus contains a significant dorsal diverticulum that passes between the ophthalmic and maxillomandibular canals. This condition is shared with *Gorgosaurus* and CMNH 7541 but is lacking in *Daspletosaurus* and *Tyrannosaurus* [21]. The basisphenoid recess pneumatizes the basipterygoid processes and retains the plesiomorphic caudal fossa that is highly reduced or absent in *Tyrannosaurus* and *Tarbosaurus* [16], [21], [39]. The subsellar sinus invests the entire length of the cultriform process of the parasphenoid so that its three-dimensional reconstruction reflects the shape of the process nearly perfectly. This sinus is restricted to the proximal base of the cultriform process in *Tyrannosaurus*, whereas it extends distally into the process in CMNH 7541, *Gorgosaurus*, and now IGM 100/1844. The subcondylar sinus of IGM 100/1844 is like that of CMNH 7541 and differs from the condition in both *Gorgosaurus* and *Tyrannosaurus* in being confluent with the rostral tympanic sinus and joining the caudal tympanic sinus to pneumatize the crista tuberalis [21].

## Discussion

### Character transformations

The internal braincase anatomy of *Alioramus altai* preserves a number of derived features that strengthen its diagnosis as both a tyrannosauroid and a unique species. We note, however, that the braincase in the only other species of *Alioramus*, *A. remotus* [40], is not well described nor has it been CT scanned. The unique neurocranial features of *A. altai* may therefore diagnose a monophyletic *Alioramus*. The presence of a reduced midbrain angle, a long, slender, and straight cochlear cavity, a gasserian ganglion positioned fully within the endocranial cavity, and a prootic fossa accepting the external foramina of the maxillomandibular canal and at least a branch of the facial canal are all characters present in IGM 100/1844 that optimize as tyrannosauroid synapomorphies based on the small number of tyrannosauroid taxa whose endocranial anatomy has been studied to date [14], [16], [21], [22], [26], [27], [31]. Whether these characters are diagnostic of Tyrannosauroidea or some more exclusive tyrannosauroid clade depends on the unstudied internal braincase anatomy of phylogenetically basal tyrannosauroids, such as *Dilong* and *Guanlong* [6], [32], [33]. The degree to which the subsellar sinus pneumatizes the cultriform process, the absence of an independent path of the rostral middle cerebral vein through the laterosphenoid, the presence of an independent medial path of the glossopharyngeal nerve, and the ramification of the facial canal within the ossified otic capsule are all unique characters that further support the status of *A. altai* as a new species. The last two autapomorphies are currently unambiguously unique to *A*. *altai* among all theropods.

IGM 100/1844 shares with other tyrannosauroids a number of endocranial features intermediate in their development between the plesiomorphic condition present in non-coelurosaurian theropods and the highly derived morphologies characteristic of non-tyrannosauroid coelurosaurs, including avialans. These include an intermediate expansion of the cerebrum (with comparable caudolateral displacement of the optic lobes), intermediate caudodorsal expansion of the rostral semicircular canal, and a slightly downturned orientation of the head. Each of these conditions can be inferred (based on an ordered character state reconstruction) to represent the ancestral coelurosaurian morphology.

Features that vary within tyrannosauroids also inform the nature of neuroanatomical transformations between non-coelurosaurian theropods and maniraptorans. Optic lobes, for example, are not visible in all tyrannosauroids (e.g., [26]) and may be obscured in the most mature specimens by a late postnatal expansion of the cranial roof and thickening of the surrounding dural envelope that may also decrease the apparent relative size of the flocculus [21]. This hypothesis is congruent with the clear optic lobes and relatively large floccular cavity of the skeletally immature IGM 100/1844. This variation suggests that a late-stage shift in developmental timing played a role in the early history of the cerebral-optic lobe configuration so exaggerated in avialans and other maniraptorans. This shift may have been a paedomorphic truncation of the expanding cranial vault and underlying dural envelope, a hypermorphic inflation of the cerebrum at the expense of the dural envelope, or some combination of the two. Regardless of the pathway, the result is a relative expansion of the cerebrum that is closely associated with increased cognition and likely played an important role in the evolutionary success of maniraptorans [15], [41].

**Figure 9:**
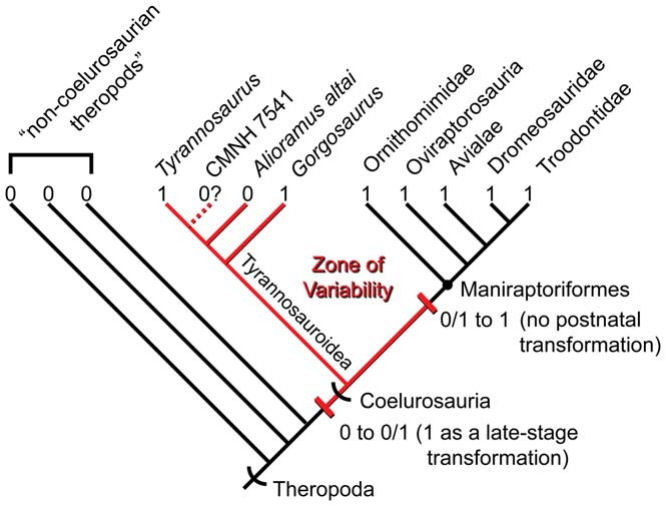
Phylogenetic tree showing the distribution of the plesiomorphic sinus cavernosus (0) in Theropoda. The sinus is invariably present in non-coelurosaurian theropods and invariably lost (1) in non-tyrannosaur coelurosaurs. The secondary loss of the sinus in *Alioramus altai* and possibly CMNH 7541 is interpreted as reflecting a period in theropod evolution in which the developmental pathways responsible for patterning the diencephalic region exhibited relative high levels of variability. This variability is retained in Tyrannosauroidea and might be expected to characterize at least the early history of its sister taxon. The variability in this case may be driven in part by heterochrony in which the original loss of the sinus occurred as a terminal addition to the ontogenetic sequence and was subsequently eliminated from postnatal ontogeny. Tree topology reflects generally accepted relationships with Theropoda [6]–[12].

The plesiomorphic features expressed in IGM 100/1844 indicate previously unrecognized evolutionary complexity and morphological plasticity in the transition from basal theropod neuroanatomy to that of maniraptorans. For example, the presence of a sinus cavernosus supports *Alioramus altai* as lying phylogenetically outside of Coelurosauria. But, when considered as a feature of the deeply nested tyrannosauroid IGM 100/1844 (and possibly CMNH 7541, which is also an ontogenetically young specimen), the presence of this sinus suggests theropod evolution went through a period at the base of Coelurosauria where the developmental pathways (genetic-epigenetic interactions during ontogeny [42]) responsible for patterning the diencephalic region exhibited a relatively high level of variability. This variability is retained to some degree in Tyrannosauroidea and can be expected to characterize at least the early history of its sister taxon (the clade of non-tyrannosauroid coelurosaurs) before becoming secondarily reduced, eventually resulting in the seemingly fixed loss of a cavernous sinus that is conserved in Aves (Fig. 9). A similar pattern can be inferred for the evolution of a straight versus strongly bowed lateral semicircular canal and a vertical versus strongly angled common crus of the osseous labyrinth.

In each of these characters, it is a transformational increase in variability at or near the base of Coelurosauria followed by a transformational reduction or loss of that variability during subsequent coelurosaurian evolution (after the divergence of tyrannosauroids) that represents the underlying synapomorphies presaging the 0 to 1 state changes reflected in phylogenetic character matrices and used to characterize broad patterns of theropod morphological evolution. In this sense, the polymorphic 0/1 condition for tyrannosauroids can be conceptualized as a derived feature that is intermediate between the 0 and 1 states of non-coelurosaurian theropods and maniraptorans, respectively. Because the 0/1 state (i.e., high variability) would only emerge at the clade level, its presence would not necessarily change the phylogenetically informative nature of 0 to 1 or 1 to 0 transformations occurring within that clade—in other words, homoplasies can be phylogenetically informative [43], [44].

The relatively young ontogenetic ages of both IGM 100/1844 and CMNH 7541 suggests that their unique expression of these plesiomorphic states may simply reflect ontogenetic variation. Modern crocodilians and birds both lack postnatal variation with regards to the presence and absence of the sinus cavernosus, respectively. The variation observed in tyrannosauroids suggests the initial loss of the sinus along the avian stem occurred as a late-stage ontogenetic transformation (terminal addition) that was subsequently pushed to earlier developmental stages. Eventually, this resulted in the absence of postnatal variation characteristic of the avian crown. Between the evolution of the derived condition as a terminal ontogenetic state and the heterochonic shift(s) that pushed this transformation out of postnatal development exists a period of increased variability where expression of the plesiomorphic and derived conditions can be expected in a typical collection of fossil specimens reflecting different stages of the growth curve.

### Concentrated homoplasy, variability, and pattern interpretation

A Darwinian model of evolution predicts that the origin of a morphological structure will generally be accompanied by a period of increased variability in the developmental pathway(s) associated with that feature. It is possible that these periods of high variability can at times be maintained long enough to influence broad patterns of phylogenetic history [45], [46]. Such extended “zones of variability” should produce distinctively high levels of homoplasy near the origin of a derived feature followed by a canalization of morphological expression. The opening and closing of these zones likely reflect phylogenetically informative transformations (i.e., 0→1, 1→2) in the molecular architecture of development that increase and subsequently decrease variability in the system (e.g., [47]).

In the transformation of the sinus cavernosus, horizontal semicircular canal, and common crus to the derived states retained in modern birds, the anatomy of *Alioramus altai* contributes to a pattern of variation that is congruent with the predicted pattern if a zone of variability was in place at the base of Coelurosauria. The phylogenetic duration of this zone is currently unclear, but the expression of the plesiomorphic morphology in specimens lying along at least the phylogenetic stem of the Maniraptoriformes clade (Ornithomimosauria + Maniraptora) should be expected. Even if an alternative evolutionary interpretation is preferred—for example, that the late-stage transformation in tyrannosauroids is a derived developmental trajectory not ancestral to those theropods more closely related to birds—the tyrannosauroid transformational pattern still establishes morphological and developmental complexity for these characters at the base of the coelurosaur tree and supports heterochrony as an important mechanism in driving patterns of neuroanatomical variability.

The extended maintenance of high variability, with its associated expectation of high levels of homoplasy, may be an important component of large-scale patterns of morphological and ecological evolution. In a study of rates of character change and morphological disparity (overall anatomical variety) in early Triassic-Jurassic archosaurian relatives of birds and crocodiles based on a 400+ discrete character dataset, the major clade of crocodile-line archosaurs (Crurotarsi or Pancrocodylia) had both significantly greater morphological disparity and a significantly higher incidence of homoplasy than bird-line archosaurs (Ornithodira or Panaves [13], including primitive theropod dinosaurs) [48]. These results suggest that homoplasy is a principal driver of large-scale morphological variation (morphospace occupation) in at least one clade of fossil vertebrates. In other words, those lineages with more homoplasy are able to occupy a greater range of morphospace and experiment with a greater number of body plans (and presumably ecological activities, if morphospace occupation roughly correlates with ecological niche occupation) than those species with less homoplasy. It may be expected, therefore, that taxa nested within an extended “zone of variability,” may be able to explore greater morphological and ecological opportunities (depending on the nature of the characters, or character complexes, affected). This tantalizing possibility awaits further testing with other empirical case studies, as well as integration of developmental, paleontological, and phylogenetic data.

The rapid rate at which our understanding of morphological evolution is progressing is largely a reflection of the data generated by developmental biologists, who are isolating the mechanisms of morphological change at the molecular level, and by paleontologists, whose unique ability to empirically study the distribution of characters in deep time is critical to establishing the tempo and mode of these mechanistic transformations in developmental pathways. The role of paleontology is especially crucial when long periods of evolutionary history separate the origins and radiations of modern groups [49]. Conceptualizing the patterns of morphological variation and homoplasy derived from anatomical studies, such as this one on *Alioramus altai*, as a reflection of evolving variability in developmental networks [44], [50]–[52] increases the potential of paleontology to inform the nature of morphological transformations and the molecular changes that underlie them. This can only strengthen the relationship between fossil and developmental data [53].

## Materials and Methods

### Specimen and CT scanning

IGM 100/1844 was collected under the auspices of an institutional agreement between the American Museum of Natural History and Mongolian Academy of Science. All specimens collected under the terms of this agreement receive Mongolian Academy catalogue numbers and are returned to Mongolia. The braincase of IGM 100/1844 was scanned at The University of Texas High-Resolution X-ray Computed Tomography Facility, Austin. Scanning occurred along the sagittal axis using a voltage of 450 kV and amperage of 3 mA. A total of 431 slices were recovered at an image resolution of 1,024 X 1,024 pixels. The slice thickness and interslice spacing is 0.25 mm with a reconstructed field of view of 301.6 mm. Reslicing of the data along the horizontal and coronal axes and all digital segmentation was performed using VG StudioMax 1.2.1. The reconstruction of all movies (www.digimorph.org/specimens/Alioramus_altai) was performed using VG StudioMax 2.0.1.
